# Current Status in Rechallenge of Immunotherapy

**DOI:** 10.7150/ijbs.82776

**Published:** 2023-05-07

**Authors:** Han Hu, Ke Wang, Rong Jia, Zi-Xun Zeng, Min Zhu, Yuan-Le Deng, Zhu-Juan Xiong, Jian-Ning Tang, Hua Xie, Yi Wang, Peng Zhang, Jin Zhou

**Affiliations:** 1 Department of Medical Oncology, Sichuan Clinical Research Center for Cancer, Sichuan Cancer Hospital & Institute, Sichuan Cancer Center, Affliated Cancer Hospital of University of Electronic Science and Technology of China, Chengdu, China.; 2 Division of Nutritional Medicine, Sichuan Clinical Research Center for Cancer, Sichuan Cancer Hospital & Institute, Sichuan Cancer Center, Affliated Cancer Hospital of University of Electronic Science and Technology of China, Chengdu, China.; 3 Department of Radiotherapy, Sichuan Clinical Research Center for Cancer, Sichuan Cancer Hospital & Institute, Sichuan Cancer Center, Affliated Cancer Hospital of University of Electronic Science and Technology of China, Chengdu, China.

**Keywords:** cancers, immune checkpoint inhibitors, rechallenge, target population, strategy, safety

## Abstract

The treatment of malignant tumors has entered the era of immunotherapy, and immune checkpoint inhibitors (ICIs) have brought significant benefits to patients. However, some patients are required to discontinue treatment with ICIs owing to factors such as disease progression and intolerable side effects. Faced with limited subsequent treatment options and complex medical needs, we searched PubMed, Embase, Cochrane library, and the NIH clinical trials database and found that ICI rechallenge could be a relevant clinical strategy. The factors that could affect the rechallenge efficacy include the patients' characteristics, therapeutic strategy selection, and the timing of treatment. Multiple factors are used to identify target population, of which clinical features and PD-L1 expression are more potential. Both single ICI rechallenge and combination therapy may have survival benefits. Patients who have tolerated initial immunotherapy well could undergo ICI rechallenge, while patients who have experienced grade 3 or higher immune-related adverse events should be carefully assessed prior to rechallenge. Interventions and the interval between two courses of ICI will clearly have an impact on the efficacy of subsequent treatment. Preliminary data evaluation supports further investigation on ICI rechallenge to identify the factors that could contribute to its efficacy.

## Introduction

The incidence and mortality of patients with malignant tumors is increasing, and the public health burden of cancer treatment is increasing accordingly 1-4. There were 18.1 million new cancer cases and 9.6 million cancer deaths worldwide in 2018 5, and the numbers increased to 19.3 million and 9.96 million, respectively, in 2020 6. Cancer is considered one of the major health threats to humans. However, cancer therapy has advanced significantly in the last few decades, including the recent development of immunotherapy, which has long-term benefits for some patients with cancer 7-9.

**Figure 1:**
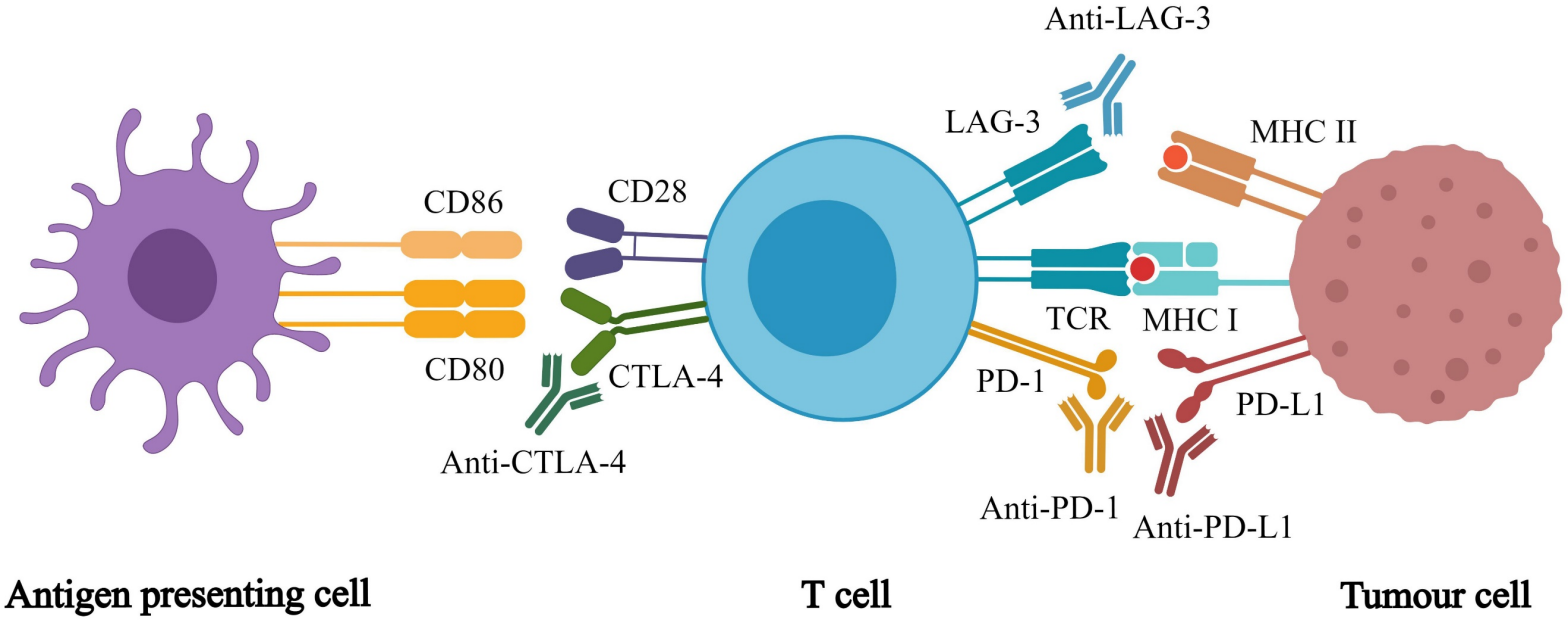
** Different immune checkpoint inhibitors and their respective targets.** Immune checkpoint inhibitors act on different targets to enhance T cell responses, which in turn promote anti-tumor effects of whole-body.

Immunotherapy enhances the immune system's tumor recognition, blocks the immunosuppressive signals from the tumor cell, and weakens the immunosuppressive nature of the tumor microenvironment 10. Compared with traditional anti-tumor therapy, immunotherapies have incomparable advantages, which can prolong progression free survival (PFS) and overall survival (OS) 11. While many studies are being conducted to discover new immunotherapies, immune checkpoint inhibitors (ICIs) are currently the most effective cancer immunotherapy 12. ICIs are monoclonal antibodies that target inhibitory immune checkpoints, which are exploited by cancer cells to escape the immune system. Approved ICIs include inhibitors of 13 programmed cell death 1 (PD-1), programmed cell death ligand 1 (PD-L1), cytotoxic T lymphocyte antigen 4 (CTLA-4), and lymphocyte activation gene 3 (Figure 1). ICI monotherapy or in combination with other anti-cancer treatments improves anti-tumor efficacy for many cancer types, which is effective in the treatment of malignant tumors 14-17. Meanwhile, immunotherapy introduces new problems and challenges. For example, ICIs only work for a few patients, and there is a lack of biomarkers to identify them. Currently, the level of PD-L1 expression is used but it has several limitations 18-19. In addition, clinicians have many unanswered questions, such as how to determine the treatment regimen and whether to use monotherapy or combination therapy. Moreover, if combination therapy is chosen, there are still questions regarding which drugs should be combined. In addition, from a clinical standpoint, how can immunotoxicity be predicted? To maximize the application of ICIs, many studies must be conducted to answer these questions.

When employing ICI therapy for tumors, we found that some patients have to discontinue ICI treatment owing to immune-related adverse events (irAEs) or progressive disease (PD). If the subsequent therapy option is limited for those patients, should or can the clinicians order another course of ICI treatment (rechallenge)? We considered this a viable option because of the long-term benefits of immunotherapy. Some studies have revealed that ICI rechallenge can be beneficial to some patients 20-27. In this review, we summarized literature data on ICI rechallenge, and hope to contribute to standardizing ICI rechallenge clinical protocols.

## Immunotherapy rechallenge/retreatment

### Definition

There are two protocols for restarting immunotherapy: retreatment and rechallenge. ICI retreatment refers to the clinical strategy whereby patients restart ICI treatment without any other cancer treatments in between 28-31, whereas the ICI rechallenge strategy involves other treatments between the two ICI courses. This is an important distinction because the additional treatments can influence the homeostasis of the patients' immune system, resulting in the need for a second course of immunotherapy. We focused on ICI rechallenge in this review because it is more broadly used 32-33.

### Significance

Cancer treatment has entered the era of immunotherapy. Patients with advanced cancers can particularly benefit from it. Nevertheless, treatment resistance and tumor progression can still happen over time. When these happen, clinical research indicates that the reuse of immunotherapy could still benefit patients, and it may be a better option than conventional cancer therapies, such as chemotherapy and radiation therapy. Immunotherapy has additional advantages such as low side effects, which allows patients to maintain a higher quality of life. Furthermore, ICIs are affordable in China, making it a viable option for many patients. On the other hand, whilst a profound number of patients are being treated with ICIs, more knowledge is needed to maximize their benefits.

### Current guidance

Current studies on ICI rechallenge are sparse. The National Comprehensive Cancer Network, the European Society for Medical Oncology, and the Society for Immunotherapy of Cancer recommend immunotherapy rechallenge for melanoma treatment; however, they have no consensus for the timing 34-37. A few guidelines recommend immunotherapy rechallenge for renal cancer and head and neck squamous cell carcinoma, but the data were insufficient to support them. Lung cancer is the second most common cancer and the leading cause of cancer death 38-39. ICI treatment significantly improved the prognosis of lung cancer in patients. However, no guidelines have been published for lung cancer ICI rechallenge. We investigated the lung cancer subgroups of recent publications on ICI rechallenge, and our findings suggest that some patients with lung cancer benefited from ICI rechallenge. Notably, the sample sizes from these subgroups were not large enough to statistically conclude the benefit of ICI rechallenge in patients with lung cancer. Based on the available data, we believe that it is worthwhile to study ICI rechallenge regarding lung cancer.

We list some of the ongoing clinical trials of ICI rechallenge in Tables 1 and 2. Most of them are Phase II clinical trials using ICI rechallenge to treat lung cancer, malignant melanoma, and urinary system tumors. The ICI rechallenge strategy includes using a single ICI, two ICIs, and an ICI combined with other anti-tumor medications. We want to point out that most studies do not focus on ICI rechallenge; rather, ICI rechallenge was used as one of the treatment options, and it was used independently or in combination therapies. Moreover, the decision whether to use other anti-tumor treatments between two courses of ICIs varied, and the primary endpoints were efficacy or safety. In other words, the information solely on ICI rechallenge is limited. In this review, we highlighted the data in an attempt to spark interest in the study of ICI rechallenge.

## The current research status

### ICI rechallenge target population

Currently, ICIs can only benefit small patient populations, and how to identify them has received increasing interest. PD-L1 expression level is the primary choice 40-41. The same applies to ICI rechallenge; however, clinicians are also aware of its limitations. No conclusion has been reached to identify patients who can benefit from ICI rechallenge, and we believe this should be a priority for ICI rechallenge clinical studies. Meanwhile, we summarize the main inclusion and exclusion criteria from the ongoing studies in Tables 3 and 4.

#### Clinical features

##### Patient's general condition

ICI treatment outcomes vary greatly among patients. The main contributing factors include Eastern Cooperative Oncology Group performance status (ECOG/PS) and nutritional status. In a retrospective study 42, 35 patients with advanced non-small cell lung cancer (NSCLC) were retreated with ICIs after pausing the initial ICI treatment, owing to disease progression. The median PFS and OS were 81 days (95% confidence interval [CI] 41-112 days) and 225 days (95% CI 106-361 days), respectively. Multivariate analysis showed that an ECOG/PS score ≥2 (hazard ratio [HR] 2.38; 95% CI 1.03-5.52; *p*=0.043) was negatively associated with PFS, while body mass index (BMI) >20 (HR 0.43; 95% CI 0.19-0.95; *p*=0.036) was positively associated with PFS. Therefore, the researchers proposed that ECOG/PS score and BMI could be evaluation factors to decide if patients should receive ICI rechallenge. In another study, Gobbini et al. 43 analyzed the outcome of ICI rechallenge in 144 patients with advanced NSCLC and found that patients with ECOG/PS score of 0, 1, and ≥2 have a median OS of NR (95% CI 2.1-not reached), 1.4 years (95% CI 0.2-2.1), and 1.1 years (95% CI 0.7-1.6), respectively. These studies suggest that patients with NSCLC who had better ECOG/PS scores (ECOG/PS ≤1) could benefit from ICI rechallenge. We suspect that this correlation is linked to treatment tolerance. Patients with better physical health can tolerate ICI rechallenge better, and the ICI efficacy is correlated with the clinical treatment duration. We need to point out that physical fitness is subjective; we would like to see clinical studies to list both patient fitness data and ICI rechallenge tolerance data.

##### Initial immunotherapy

Researchers have investigated the relationship between ICI rechallenge clinical outcomes and initial immunotherapy. Levra et al. 44 evaluated 1,517 patients who received ICI rechallenge in the French national hospital database of 10,452 patients with locally advanced or metastatic NSCLC. Between the initial ICI treatments and rechallenge, 1,127 patients did not receive any therapy, whilst 390 patients received chemotherapy. Among the 1,127 patients, Levra found that the median OS increased for patients who received the initial ICI treatment for ≥6 months (HR 0.19; 95% CI 0.14-0.25; *p*<0.0001) and 3-6 months (HR 0.56; 95% CI 0.46-0.70; *p*<0.0001) compared with patients who received the initial treatment for <3 months. Therefore, patients' ICI rechallenge outcome is positively correlated with the length of their initial treatment. One hypothesis states that longer initial immunotherapy strengthens the immune memory. Another hypothesis states that patients with longer initial treatment are the dominant population for immunotherapy. Vauchier et al. 45 analyzed the data of 45 patients with metastatic renal cell carcinoma in a multicenter study. Patients with a PFS of 6-12 months (HR 0.55; 95% CI 0.17-1.78; *p*=0.07) and >12 months (HR 0.25; 95% CI 0.08-0.84; *p*=0.07) responded better to their initial ICI treatment than patients with a PFS ≤6 months. A melanoma study with a small sample size also revealed similar results 46. In addition, Niki et al. 47 conducted a retrospective study and found that four out of five patients with advanced NSCLC who responded to ICI rechallenge also responded to their initial ICI therapy. In contrast, a retrospective study by Santini et al. 20 showed that of 482 patients with NSCLC who received ICI treatment, 20 achieved a partial response (PR) for the initial ICI therapy; however, the treatment was stopped owing to irAEs, PFS (HR 0.68; 95% CI 0.19-2.24; *p*=0.56) and OS (HR 0.37; 95% CI 0.06-2.21; *p*=0.28), which were similar between the rechallenge (*N*=12) and control (*N*=8) groups. Another study revealed a similar outcome for patients with advanced melanoma treated with nivolumab and ipilimumab 48. This suggests that the initial ICI treatment outcome cannot be used as the sole indicator to predict ICI rechallenge outcome, particularly when severe irAEs occur during treatment. Summarily, initial ICI therapy efficacy can be a good indicator for ICI rechallenge; however, it should not be the sole consideration.

##### Interruptive reasons for immunotherapy

Most initial immunotherapy was discontinued abruptly for two reasons: irAEs or PD. Reschke et al. 49 analyzed the data of 570 patients with advanced melanoma from different studies who paused their initial ICI treatments and found that the most common cause as PD (381/570, 67%), followed by severe irAEs (189/570, 33%). Patients (*N*=85) who received anti-PD-1 antibody rechallenge following PD during their initial anti-PD-1 therapy had a mean disease control rate (DCR) of 45.8%, mean objective response rate (ORR) of 15.5%, and mean PFS of >8.2 months. Patients (*N*=114) who received anti-PD-1 and anti-CTLA-4 antibody rechallenge had a mean DCR of 40.6% and a mean ORR of 20%. In addition, patients (*N*=182) rechallenged with anti-CTLA-4 antibody following PD during their initial anti-CTLA-4 therapy had a mean DCR of 50.9% and a mean ORR of 20.4%. Meanwhile, patients (*N*=189) who received anti-PD-1 antibody rechallenge following toxicity-related treatment discontinuation had a mean DCR of 89.5%, a mean ORR of 70.2%, and a mean PFS of >7.4 months. This indicates that ICI rechallenge could benefit patients who paused their initial immunotherapy regardless of the cause. Two separate cohorts have a significantly different DCR and ORR but a similar PFS. The reason behind this phenomenon is worth exploring. The meta-analysis by Inno et al. 50 found similar outcomes. The overall response rate of ICI rechallenge was 21.8% regardless of the reason for pausing the initial treatments. This is important because it shows that ICI rechallenge could translate into survival benefits for patients who did not finish their initial course of ICI treatment. From the data, we hypothesize that ICI rechallenge might restore some treatment benefits that were lost owing to the incompletion of the initial treatment. Sheth et al. 51 provided partial data for patients (*N*=168) with advanced solid tumors who completed 1 year of initial durvalumab treatment and then restarted (*N*=70) after tumor relapse. More than half of the 70 patients achieved disease control (eight patients had PR, 42 had stable disease [SD]), and four had ≥ grade 3 irAEs. The retrospective study by Gobbini et al. 43 agrees with these outcomes. The PFS (irAE: HR 0.54, 95% CI 0.33-0.86; clinical decision: HR 0.63, 95% CI 0.37-1.07) and OS (irAE: HR 0.44, 95% CI 0.23-0.82; clinical decision: HR 0.61, 95% CI 0.23-0.82) of rechallenged patients who were suspended prior to ICI rechallenge owing to irAEs or clinical decisions were prolonged compared with those with PD. Taken together, the reasons for pausing the initial ICI treatment might affect the rechallenge outcome; however, there are different tendencies. If patients discontinued ICI therapy owing to irAEs, clinicians should try to design treatment regimens to avoid adverse reactions and allow patients to receive therapy for as long as possible. If patients discontinued owing to PD, different ICIs for rechallenge should also be considered, although choosing depends on more clinical trials.

#### Biomarkers

Immunotherapy still lacks effective biomarkers to predict efficacy. Based on clinical studies, including KEYNOTE-010, KEYNOTE-024, and CheckMate026, PD-L1 expression level and tumor mutation burden (TMB) are the most commonly used biomarkers 52-54, and the former is frequently used 55. ICI rechallenge also lacks biomarkers, and clinicians try to use PD-L1 and TMB as substitutes. Regrettably, retesting for PD-L1 before rechallenge is still rarely achieved in the real-world. A retrospective study examined 12 patients with NSCLC who were administered pembrolizumab rechallenge after pausing initial nivolumab treatment; the authors found that all patients who showed responses (PR and SD) had high PD-L1 expression (tumor proportion score ≥80%) 56. However, a different study analyzed 35 patients with NSCLC from six Japanese institutions who received ICI rechallenge and did not find the rechallenge efficacy to be correlated with PD-L1 expression level 42. Therefore, it is unclear whether the PD-L1 expression level could reliably predict the outcome of ICI rechallenge. The PD-L1 expression level in many retrospective studies was only determined during the initial ICI treatment, and we do not know if the PD-L1 expression level changes following the initial immunotherapy. We believe it is important to measure before the rechallenge.

The anti-tumor immune responses are heavily influenced by the microenvironment. Therefore, researchers studied whether inflammatory indicators, such as neutrophil to lymphocyte ratio (NLR), lymphocyte to monocyte ratio (LMR), and platelet to lymphocyte ratio (PLR), could predict immunotherapy outcomes 57-59. The aforementioned study of patients with NSCLC 42 found that PFS from ICI rechallenge correlated with a NLR ≥5 and a LMR <1.7, and the OS correlated with a PLR >262. Kan et al. 60 found that patients with advanced melanoma who achieved a PR showed an increase in the NLR during initial immunotherapy but a decrease after sequential non-ICI treatment, whereas the NLR remained unchanged in patients who did not respond. A change in the NLR might result from a fluctuating tumor microenvironment. Some inflammatory indicators may be used as reference elements for the ICI rechallenge efficacy; however, the mechanisms are unclear.

### Rechallenge strategies

#### Single ICI rechallenge

No conclusion has been reached on whether ICI rechallenge should use the initial regimen or switch to other ICIs. Chiarion et al 61 examined 855 patients with advanced melanoma treated with ipilimumab, an anti-CTLA-4 antibody, and 51 patients underwent ipilimumab rechallenge after disease progression. Overall, 55% of the rechallenged patients achieved disease control (two cases of complete response, four cases of PR, and 22 cases of SD), and the median OS of the rechallenged group was significantly prolonged compared with the control group (21 months vs. 13 months, *p*<0.0001). Yang et al. 62 analyzed 22 prospective studies using ipilimumab and identified three studies (204 cases) that have ipilimumab monotherapy rechallenge data. The ORR was 12-23%, DCR was 48.4-67.7%, and median OS was 12 months. In a similar analysis of 13 studies using PD-1/PD-L1 inhibitors, six of them applied the initial drug to rechallenge, and the ORR and DCR were 11.4-53% and 47.1-83%, respectively. Several other independent studies found similar outcomes 63-64. These data indicate that ICI rechallenge using the original regimens of CTLA-4 or PD-1/PD-L1 inhibitors can benefit patients. Furthermore, other studies found that it was safe and effective to switch to different PD-1/PD-L1 inhibitors 65-67; however, their outcomes were inconsistent. Kitagawa et al. 22 retrospectively examined 17 patients with advanced NSCLC who were rechallenged with different PD-1/PD-L1 inhibitors, and 10 (58.8%) achieved a PR or SD. Watanabe et al. 68 reported that switching to different PD-1/PD-L1 inhibitors did not achieve clinical benefits. Martini et al.'s 69 data for two patients also revealed similar outcomes. Rechallenge using different ICIs could in theory achieve efficacy while reducing irAEs, and the inconsistency might be from the small sample size, which introduced large variations.

The current dominant types of ICIs are anti-CTLA-4 and anti-PD-1/PD-L1 antibodies, and they work through different biological pathways 70. The CTLA-4 pathway restricts immune responses in the early stages of T cell activation, while the PD-1/PD-L1 pathway mainly limits T cell activities in the tumor microenvironment. Switching between the two for ICI rechallenge has drawn some interest 71. Larkin et al. 72 compared patients with ipilimumab-refractory melanoma who received nivolumab or chemotherapy. They found that the former had a higher overall response rate (27% vs. 10%), longer median duration of response (31.9 vs. 12.8 months), and fewer grade 3 and 4 treatment-related adverse events (14% vs. 34%). However, the survival rates showed no difference. In the aforementioned meta-analysis by Yang et al. 62, patients with PD during the initial anti-CTLA-4 treatment had higher ORR when rechallenged with anti-PD-1 antibodies (22-36%) compared with anti-CTLA-4 antibodies (12-23%). Only two studies in this meta-analysis evaluated the efficacy of anti-CTLA-4 antibodies after PD from the initial anti-PD-1 antibody treatment. One did not observe an objective response after a median follow-up of 21.2 months; the other study reported an ORR of 22.4%. This meta-analysis revealed that anti-CTLA-4 antibody monotherapy for rechallenge has limited efficacy; therefore, anti-PD-1/PD-L1 antibodies should be used (either as monotherapy or in combination). We speculate that the systematical stimulation of T cells (through anti-CTLA-4 inhibitors) is insufficient; therefore, resolving the immunosuppression of the tumor microenvironment is also needed. In addition, anti-CTLA-4 and anti-PD-1 antibodies have different irAE profiles 73. Clinicians could consider switching provided that the treatment efficacy is not sacrificed.

#### Combination therapy

##### Combination of ICIs

The approved ICIs activate the immune system through different mechanisms, and the US Food and Drug Administration has already approved several ICI combination treatments 74-78. Glutsch et al. 79 described five patients with Merkel cell carcinoma refractory to avelumab that benefited from nivolumab and ipilimumab combination rechallenge after PD. Other studies reported similar outcomes for different cancers 80-81, although they were based on individual case studies. Silva et al. 82 examined 355 patients with PD-1/PD-L1-resistant advanced melanoma at 15 institutions for a median follow-up period of 22.1 months. Patients rechallenged with an anti-PD-1 antibody in combination with ipilimumab had longer OS (HR 0.50; 95% CI 0.38-0.66; *p*<0.0001), longer PFS (HR 0.69; 95% CI 0.55-0.87; *p*=0.0019), and higher ORR (31% vs. 13%, *p*<0.0001) than those rechallenged with ipilimumab monotherapy. In this study, patients rechallenged with a single or multiple ICIs had a similar ratio of grade 3 and 5 adverse events (31% vs. 33%). These data suggest that ICI rechallenge involving the combination of different types of ICIs was superior to using a single ICI. Studies on other cancers showed similar outcomes 83-85; however, they lack a control group using a single ICI. Moreover, Reschke et al. 49 found that anti-PD-1 plus anti-CTLA-4 antibody dual rechallenge regimen (mean DCR: 40.6%, mean ORR: 20%) was not superior to anti-PD-1/PD-L1 monotherapy for patients who relapsed after the initial anti-PD-1 therapy. Zimmer et al. 86 found a similar outcome where 1-year OS rates were 54% and 55% for dual and mono ICI rechallenge, respectively. In addition, Ravi et al. 87 found that more patients achieved a PR or SD with mono-ICI rechallenge (*N*=26) than with dual-ICIs rechallenge (*N*=22). Summarily, dual-ICIs rechallenge which activates the anti-tumor immune response from different angles remains a fascinating concept; however, the outcome was mixed. Further large, multi-center sample studies may help to explain the findings.

##### Combining with non-ICI anti-cancer therapies

Chemoradiotherapy and targeted therapy are still the dominant anti-cancer treatments 88-89. Several clinical studies are being conducted to combine these treatments with immunotherapies. This could potentially expand the patient population that can benefit from immunotherapy, which remains low. Several studies 90-91 have reported that adding chemotherapy or anti-angiogenic drugs to ICI rechallenge showed objective and durable tumor responses. A phase II study 92 assessed patients with metastatic renal clear cell carcinoma who had PD during the initial ICI treatment and were rechallenged with the combination of pembrolizumab and levatinib (an anti-VEGF inhibitor). Half of the patients had an objective response (ORR: 51%). Moreover, ICIs combined with mitogen-activated protein kinase kinase inhibitors, gonadotropin-releasing agents, and inhibitors of apoptosis protein antagonists are also being actively explored in clinical trials as second-, third- or fourth-line treatments 93-97. Lemaire et al. 98 used the quantitative scoring methodology to help screen drugs and further discovered combinations with potential for success. This provides a novel and promising idea for future studies and utilization of ICI rechallenge. Selected ongoing clinical trials for such combination are presented in Table 5. One more issue to take into account is that the differences in drug dosages between initial immunotherapy and ICI retreatment/rechallenge lack an integrative systematic analysis. And there are also few related studies on the dose-dependent actions of subsequent ICI. But it might be worth a word to mention that the dose of subsequent ICI was essentially the same as the initial treatment from a clinical perspective. The optimal dose still requires further exploration.

### Safety management

Immunotherapy is a new field compared with chemo- and radiation therapies in which the tolerance profile is largely under-explored. The safety concerns are particularly important for patients who paused the initial ICI treatments owing to irAEs 99-100. Fujisaki et al. 101 showed that patients who paused their initial ICI treatment owing to irAEs could tolerate ICI rechallenge and also achieved improved OS (*p*=0.025). Bhatlapenumarthi et al. 102 identified 27 patients who received ICI rechallenge in their retrospective analysis of 465 patients with advanced solid tumors, of which 18 patients showed good tolerance (18/27). A cohort study conducted by Dolladille et al. 21 observed the recurrence rate of irAEs to be 28.8% (95% CI 24.8-33.1) for ICI rechallenge. Simonaggio et al. 103 examined rechallenged patients who experienced ≥ grade 2 irAEs during the initial ICI treatment. They found that 22 patients (55%) experienced the recurrence of irAEs; however, none of them had irAEs that were more severe than the initial ones, and the irAE onset time was delayed compared with the initial treatment (9.15 weeks vs. 15 weeks, *p*=0.04). Several other studies have shown that ICI rechallenge was better tolerated, even for patients who paused the initial treatments owing to irAEs 68, 104-107. However, not all studies revealed this trend. Zhao et al. 108 performed a meta-analysis that included 789 cases from 18 cohort studies and five case series. Their findings revealed that ICI rechallenge produced equal incidences of ≥ grade 3 irAEs (*p*>0.05) but higher incidence in other categories (OR=3.81; 95% CI 2.15-6.74; *p*<0.0001). Pollack et al. 109 examined patients with advanced melanoma who paused the initial anti-PD-1 plus anti-CTLA-4 antibody regiment owing to irAEs and were then rechallenged with anti-PD-1 antibodies. Approximately 50% of them had irAEs; however, whilst most were low-grade, life-threatening cases were also observed. They also discovered that the severity of irAEs from the initial treatments could not predict the outcome of the rechallenge (*p*=0.9). Particularly, clinicians should pay attention to the cardiac toxicities, neurological toxicities, and any grade 4 irAEs during the rechallenge regardless of the initial adverse effects 110-112. Some scholars believe clinicians should be cautious about applying ICI rechallenge to patients who could benefit from rechallenge but suffer from grade 3 or higher irAEs. The cost-benefit should be carefully balanced 113-114. Currently, we cannot predict either the efficacy or irAEs of ICI rechallenge. Managing immunotoxicities should, therefore, be a high priority for clinicians, especially for patients who experienced ≥ grade 3 irAEs 115-116. We listed partial safety data from the ICI rechallenge in Table 6.

### Rechallenge timing

#### Interval between two ICI courses

In the real-world clinical setting, a treatment interval should exist between the initial treatment and ICI rechallenge. If the rechallenge is performed too soon, the ICIs from the initial treatment could still exist in the patients' blood circulation because some ICIs have long half-lives 123-125. A sustained drug effect could keep tumor cells in a dormant state, and tumor progression was inhibited by immune mechanisms. Additionally, the time from immune induction to tumor death varies between individuals; therefore, the length of the intervals may also affect ICI rechallenge outcome. Cybulska et al. 126 retrospectively examined 116 patients with advanced melanoma who were rechallenged with ipilimumab after the initial anti-PD-1 antibody treatment. The interval varied and the medium length was 4 weeks. They did not find a correlation between the length of the intervals with median PFS (*p*=0.12), median OS (*p*=0.938), or irAEs from the rechallenge (*p*=0.7403). Fujisawa et al. 127 discovered a similar outcome (*p*=0.32), and the incidence of irAEs was similar between the patients with intervals ≤28 days and >28 days. Patients with longer intervals had a significantly reduced incidence of ≥3 types of irAEs (3% vs. 25%, *p*=0.013). The mechanism of action of the subsequent ICI could differ from the initial ICI, which could persist in the patients' blood circulation; thus producing combinational therapy-like effects 128. When such patients could not be distinguished from rechallenged patients after long intervals, the findings of such investigations should be scrutinized. NiKi et al. 47 retrospectively examined 11 patients with advanced NSCLC who received ICI rechallenge using the initial ICI. They found that patients who responded to rechallenge had a shorter treatment interval than those who did not (1.6 vs. 4.7 months), suggesting that the immunological memory from the initial treatments could last after the treatment ends. Additionally, when the immune response from the initial treatment is weakened, the originally established tumor dormancy may be broken which in turn loses the control of tumor growth, and rebuilding it might take substantial effort 129. Therefore, the researchers recommended rechallenging within 3 months. In conclusion, the interval between two courses of ICI may affect the rechallenge efficacy. The maintenance of tumor dormancy during immune balance is indispensable, and drug half-lives and immunological memory are believed to be contributing factors.

#### Interventions during immunotherapies

Immunotherapies stimulate immune responses through tumor-specific neoantigens, which can be influenced by other antineoplastic therapies, such as chemotherapy, radiotherapy, targeted therapy, and anti-vascular therapy 130. An active endogenous anti-tumor immune response induces and maintains tumor dormancy under continued action, and traditional therapies can reinvigorate debilitating endogenous immune response pathways by enhancing tumor immunogenicity 131-132. Consequently, interventions before ICI rechallenge could impact its efficacy. Some potential pathways include 133-138: increasing the expression of major histocompatibility complex class I antigen on tumor cells, recruiting more antigen-presenting cells, changing the tumor immunosuppressive microenvironment or up-regulating PD-L1 expression on tumor cells, and inducing cytotoxic effects to promote the release of tumor antigens and damage-associated molecular patterns (DAMPs) therefore reactivating the cancer-immune cycle. ICI rechallenge after interventions revealed an increased immune response in several small sample studies on patients with advanced melanoma and NSCLC 139-140. Watanabe et al. 68 included 14 patients who received ICI rechallenge in their retrospective study of 434 patients with advanced NSCLC. Of the three cases achieving disease control (PR=1, SD=2), two received radiotherapy before rechallenge. Reinhorn et al. 141 reported 45 patients with advanced NSCLC who received ICI rechallenge; nine underwent local radiotherapy for oligometastases, while another had active adrenal metastases and underwent surgery before the rechallenge. Patients who received interventions (92% vs. 58%, *p*=0.17) achieved better disease control compared with the ones who did not (31% vs. 58%, *p*=0.008). Some researchers also believe that ablation therapy could stimulate the local immune system 142. Wei et al. 143 described two patients with NSCLC who discontinued combination therapy of camrelizumab with ablation therapy owing to severe immune-related pneumonia (G2-G3). They achieved a PR after retreatment. In another study, four patients with advanced melanoma 60 were administered dacarbazine between the initial nivolumab treatment (paused owing to PD) and pembrolizumab rechallenge, and two patients achieved a PR. Notably, dacarbazine may have other advantageous effects on the immune system. Tedbirt et al. 144 had similar findings. Cabezas et al. 145 described one patient with squamous cell carcinoma of the head and neck who paused the initial PD-L1 treatment owing to PD and achieved a PR after receiving paclitaxel followed by nivolumab rechallenge. Levra et al. 44 found that patients who underwent chemotherapy between two lines of ICI treatments had a median OS of 18.1 months (95% CI 14.6-21.6), while those who did not had a median OS of 14.8 months (95% CI 13.4-16.5). Unfortunately, not all studies reached the same conclusion. Gobbini et al. 43 found that patients who underwent chemotherapy before ICI rechallenge had a shorter PFS (HR 1.81; 95% CI 1.21-2.72; *p*=0.004) and OS (HR 1.52; 95% CI 0.90-2.60; *p*=0.1). Similar outcomes (PFS: 6.6 vs. 2.1 months, *p*=0.001) were reported by another retrospective analysis 146. Vauchier et al. 45 reported 26 patients who received therapies (nine of whom received more than one) before the ICI rechallenge and their PFS was shorter. In conclusion, interventions between two lines of ICI treatments have two contrary effects. It could stimulate exhausted tumor-specific T cells in the immune system to maintain tumor dormancy and also weaken the patients' body condition thereby dampening the immune system 147. Not all therapies may be useful before the rechallenge. A prospective study to identify the drugs or treatments that can improve ICI rechallenge efficacy may be warranted.

## Conclusion and Perspectives

With the growing advent of new ICIs, many patients could benefit from them. The potential of ICIs in the field of tumor treatment has attracted increased attention, and new therapeutic strategies are continually being explored to gain better oncotherapy efficiency. In this review, we assess the current status of ICI rechallenge and recognize that patients could benefit from this treatment paradigm. Many factors can increase the complexity of the treatment and influence the outcomes of ICI rechallenge, such as patients' clinical and pathological features, different rechallenge strategies, length of treatment pause interval, and additional treatments before the rechallenge. However, most clinical studies identified were based on a retrospective analysis of a subset of other immunotherapy clinical studies; hence, some aforementioned factors were not included in the data. In addition, the number of patients in some studies were low and produced contradictory outcomes among different publications.

Based on the effective survival benefit, ICI rechallenge is one promising way to release the underexplored potential of immunotherapy. Future studies are needed to address the following questions: How can patients who are most likely to benefit from ICI rechallenge be identified? How should the optimal rechallenge treatment regimen for the target population be selected? How can the therapeutic efficacy and safety of ICI rechallenge be maximized while minimizing adverse effects? These findings will help to establish a standardized treatment regimen, which can be applied in routine clinical practice.

## Figures and Tables

**Figure 2:**
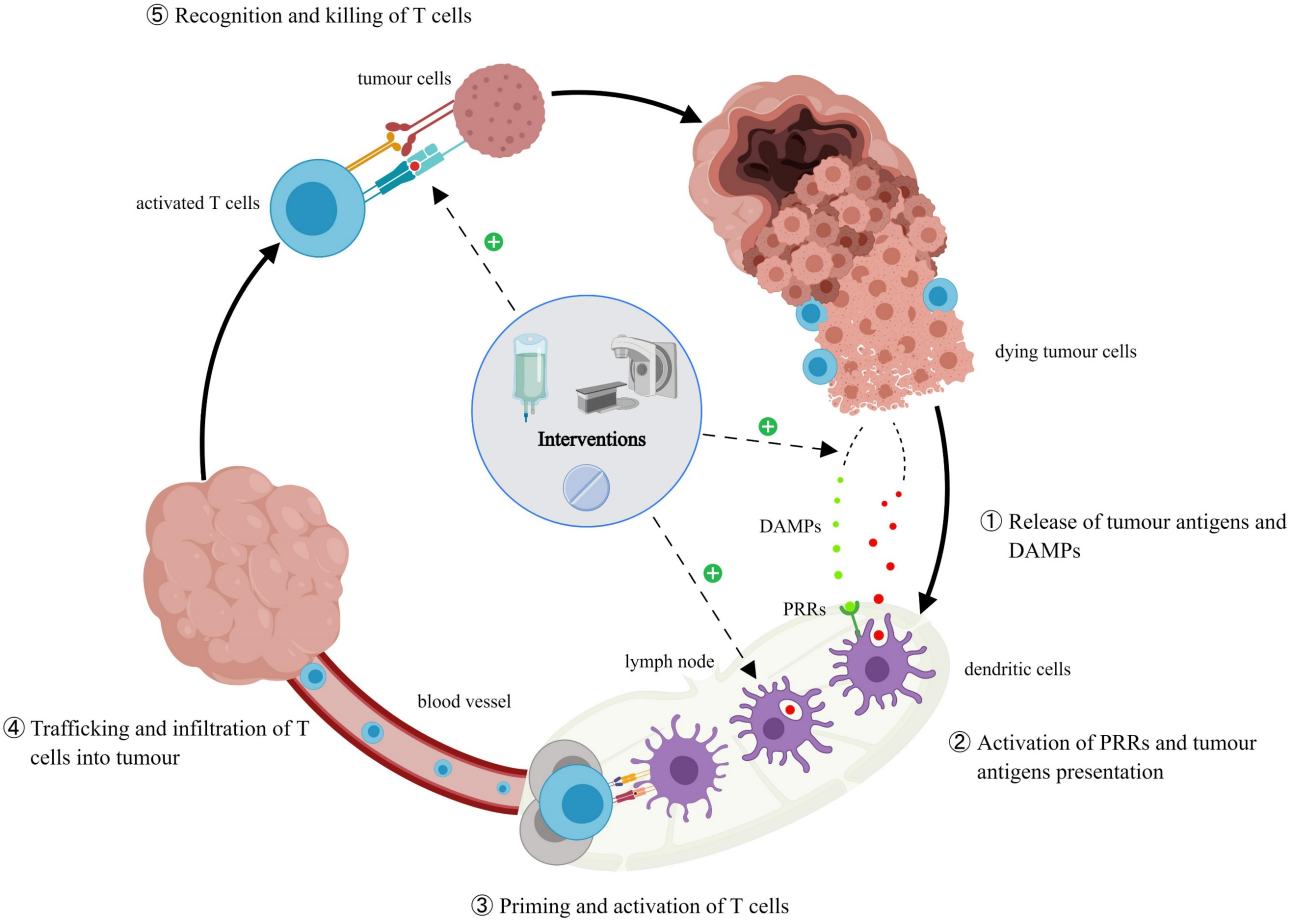
** The cancer immunity cycle and the effects of interventions.** Additional interventions promote the release of tumor antigens and DAMPs in the host, increase the number of antigen-presenting cells and their antigen presentation ability, induce the expression of major histocompatibility complex class I antigen, and change tumor immunosuppressive microenvironment and PD-L1 expression, thus having an impact on ICI rechallenge.

**Table 1:** Ongoing ICI rechallenge clinical trials related to lung cancer

Cancer type	Prior ICI	Rechallenge regimen	Endpoints	Phase	Trial
NSCLC	Nivolumab+Ipilimumab	Nivolumab+Ipilimumab	PFS	III	NCT03469960
NSCLC	ICI	Nivolumab+Anlotinib	ORR	Ib/IIa	NCT04507906
NSCLC	Anti-PD-1	Atezolizumab+platinum doublet chemotherapy	ORR	II	NCT03977467
NSCLC	ICI	Atezolizumab+Tocilizumab	ORR	Ib/II	NCT04691817
NSCLC	ICI	Atezolizumab+Ramucirumab	ORR	II	NCT03689855
NSCLC	Anti-PD-(L)1	Camrelizumab+Apatinib	PFS	II	NCT04670913
NSCLC	Anti-PD-(L)1	Camrelizumab+famitinib	OS	III	NCT05106335
NSCLC	Anti-PD-(L)1	Pembrolizumab	ORR	II	NCT03526887
NSCLC	Anti-PD-(L)1	Pembrolizumab+Docetaxel/Pemetrexed/Gemcitabine	PFS	II	NCT03083808
NSCLC	Anti-PD-(L)1	Durvalumab	ORR	II	NCT03334617
SCLC	Anti-PD-(L)1	Durvalumab+Topotecan hydrochloride	OS	II	NCT04607954

**Table 2:** Ongoing ICI rechallenge clinical trials related to other cancers

Cancer type	Prior ICI	Rechallenge regimen	Endpoints	Phase	Trial
Melanoma	Anti-PD-(L)1	Pembrolizumab+Ipilimumab	ORR	II	NCT02743819
Melanoma	Anti-PD-1±Ipilimumab	Pembrolizumab+4SC-202	safety	Ib/II	NCT03278665
HCC	ICI	Camrelizumab+Apatinib	ORR	II	NCT04826406
HCC	ICI	Sintilimab+Lenvatinib	ORR	II	NCT05010681
HCC	Anti-PD-(L)1	Pembrolizumab+Regorafenib	ORR	II	NCT04696055
GC/CRC	Anti-PD-(L)1	Tislelizumab+Anlotinib	ORR	II	NCT04777162
UC	ICI	Same ICI	Efficiency	II	NCT04322643
UC	Anti-PD-(L)1	Atezolizumab+Carboplatin+Gemcitabine	PFS	II	NCT03737123
TCC	ICI	Pembrolizumab+Ramucirumab	ORR	II	NCT04179110
NPC	Anti-PD-(L)1	Sintilimab+IBI310	ORR	Ib/II	NCT04945421
RCC	Nivolumab	Nivolumab+Ipilimumab	ORR	II	NCT03177239
RCC	Nivolumab+Ipilimumab	Nivolumab+Ipilimumab	ORR	II	NCT03126331
RCC	Nivolumab+Ipilimumab	Nivolumab+Ipilimumab	DCR	II	NCT04088500
RCC	Anti-PD-(L)1	Atezolizumab+Cabozantinib	PFS/OS	III	NCT04338269
SCCHN	Anti-PD-1	Pembrolizumab+Radiation	ORR	II	NCT03085719
Solid tumor	Durvalumab	Durvalumab	safety	II	NCT03847649
Solid tumor	Anti-PD-(L)1	Pembrolizumab+BI 1206	safety	I/IIa	NCT04219254

**Table 1 and Table 2** NSCLC, non-small-cell lung cancer; SCLC, small-cell lung cancer; HCC, hepatocellular carcinoma; GC, gastric cancer; CRC, colorectal cancer; UC, urothelial carcinoma; TCC, transitional cell carcinoma; NPC, nasopharyngeal carcinoma; RCC, renal cell carcinoma; SCCHN, squamous cell carcinoma of the head and neck; ICI, immune checkpoint inhibitor; ORR, objective response rate; DCR, disease control rate; PFS, progression free survival; OS, overall survival.

**Table 3:** The main inclusion and exclusion criteria from the ongoing clinical trials on lung cancer

Trial	Main inclusion criteria				Main exclusion criteria			
	Cancer type	ECOG/KPS	PD-L1	Prior treatment	irAE	Metastases	Driver mutation	Other
NCT03469960	stage IV NSCLC	0-1	≥1%	PD on/after prior ICI	Grade ≥3	active untreated brain	EGFR/ALK/ROS1/HER	-
NCT04507906	stage IIIB/IIIC/IV NSCLC	0-1	-	PD on/after prior ICI	Grade ≥3	active untreated brain	EGFR/ALK/ROS1/unknown	obvious hemorrhage symptom
NCT03977467	advanced NSCLC	0-2	≥1%	PD after initial disease control of prior ICI	Grade ≥2	active untreated brain	EGFR/ALK/ROS1	uncontrolled tumor-related pain
NCT04691817	stage IV or recurrent NSCLC	0-2	-	PD on/after prior ICI	Grade ≥3	active untreated brain	susceptible to targeted therapy	uncontrolled tumor-related pain
NCT03689855	NSCLC	0-1	-	PD on/after prior ICI	Grade ≥3	active untreated brain	EGFR/ALK but not treated	-
NCT04670913	stage IV or recurrent NSCLC	0-1	-	SD ≥3 months with the first immunotherapy	Grade ≥2	active untreated brain	EGFR/ALK	squamous cell NSCLC
NCT05106335	metastatic or recurrent NSCLC	0-1	-	PD on/after prior anti-PD-(L)1	-	-	-	uncontrolled pleural effusion or ascites
NCT03526887	recurrent NSCLC	0-1	≥1%	PD >12 weeks after the last dose of prior ICI	Grade ≥3	active untreated brain	EGFR/ALK	-
NCT03083808	stage IV NSCLC	0-1	-	PFS ≥3 months with the first immunotherapy	Grade ≥2	active untreated brain	EGFR/ALK/ROS1 but not treated	-
NCT03334617	metastatic or recurrent NSCLC	0-1	-	PD on prior anti-PD-(L)1	-	symptomatic brain	EGFR/ALK/ROS1/BRAF/MET/RET	-
NCT04607954	SCLC	0-1	-	PD after prior anti-PD-(L)1	Grade ≥3	active untreated brain	-	uncontrolled intercurrent illness

**Table 4:** The main inclusion and exclusion criteria from the ongoing clinical trials on other cancers

Trial	Main inclusion criteria				Main exclusion criteria			
	Cancer type	ECOG/KPS	PD-L1	Prior treatment	irAE	Metastases	Targeted therapy	Other
NCT02743819	advanced melanoma	0-1	-	SD ≥24 weeks with first-line anti-PD-(L)1	-	active untreated brain	-	active pneumonitis or active infection
NCT03278665	unresectable stage III/IV melanoma	-	-	non-responding to prior anti-PD-1	-	symptomatic brain	-	CR or PR on/after prior ICI
NCT04826406	HCC	0-1	-	PD after 2 cycles of prior ICI	intolerable	central nervous system	Apatinib	hepatic encephalopathy or symptomatic ascites
NCT05010681	unresectable or metastatic HCC	0-2	-	PD on/after prior anti-PD-(L)1	-	central nervous system	Lenvatinib	gastrointestinal bleeding
NCT04696055	unresectable advanced HCC	0-1	-	SD ≥8 weeks with first-line anti-PD-(L)1	Grade ≥2	active central nervous system	Regorafenib	pleural effusion or ascites
NCT04777162	unresectable or metastatic GC/CRC	0-1	≥1%	CR or PR with first-line anti-PD-(L)1	severe	brain	Anlotinib	uncontrolled pleural effusion
NCT04322643	advanced or metastatic UC	≥70	-	SD ≥24 weeks with the first immunotherapy	-	-	-	serious medical condition
NCT03737123	metastatic or unresectable UC	0-2	-	PD after prior anti-PD-(L)1	Grade ≥2	active central nervous system	-	-
NCT04179110	metastatic or unresectable TCC	0-1	-	PD on/after prior ICI	-	brain	VEGF/VEGFR targeting drug	uncontrolled pleural effusion or ascites
NCT04945421	metastatic or recurrent NPC	0-1	-	Failed to prior Anti-PD-1 resistance	-	-	-	uncontrolled life-threatening illness
NCT03177239	unresectable or metastatic RCC	0-1	-	PD on/after prior ICI	-	untreated brain	-	-
NCT03126331	advanced or metastatic RCC	≥70	-	CR, PR or SD after 24 weeks of nivolumab	-	active untreated brain	-	serious medical condition
NCT04088500	advanced RCC	-	-	PD of maintenance treatment of nivolumab	-	active central nervous system	-	-
NCT04338269	advanced or metastatic RCC	≥70	-	PD on/after prior ICI	-	active untreated brain	Cabozantinib	uncontrolled pleural effusion or ascites
NCT03085719	metastatic SCCHN	0-1	-	CR, PR or SD after 6 cycles of anti-PD-1	intolerable	active untreated brain	-	uncontrolled intercurrent illness
NCT03847649	advanced solid tumor	0-1	-	SD ≥8 weeks with first-line durvalumab	Grade ≥3	symptomatic brain	-	-
NCT04219254	advanced solid tumor	0-1	-	PD <12 weeks after prior anti-PD-(L)1	Grade ≥3	active central nervous system	-	serious medical condition

**Table 3 and Table 4** NSCLC, non-small-cell lung cancer; SCLC, small-cell lung cancer; HCC, hepatocellular carcinoma; GC, gastric cancer; CRC, colorectal cancer; UC, urothelial carcinoma; TCC, transitional cell carcinoma; NPC, nasopharyngeal carcinoma; RCC, renal cell carcinoma; SCCHN, squamous cell carcinoma of the head and neck; ECOG, Eastern Cooperative Oncology Group; KPS, Karnofsky Performance Status; PD-1, programmed cell death 1; PD-L1, programmed cell death ligand 1; CR, complete remission; PR, partial remission; SD, stable disease; PD, progressive disease; ICI, immune checkpoint inhibitor; PFS, progression free survival; irAE, immune-related adverse event.

**Table 5:** Ongoing clinical trials of combination therapy

Combination therapy	Cancer type	Rechallenge regimen	Endpoints	Phase	Trial
ICI+ICI	NSCLC	Nivolumab+Ipilimumab	PFS	III	NCT03469960
	RCC	Nivolumab+Ipilimumab	ORR	II	NCT03177239
	RCC	Nivolumab+Ipilimumab	DCR	II	NCT04088500
	RCC	Nivolumab+Ipilimumab	ORR	II	NCT03126331
	Melanoma	Pembrolizumab+Ipilimumab	ORR	II	NCT02743819
	NPC	Sintilimab+IBI310 (Anti-CTLA-4)	ORR	Ib/II	NCT04945421
ICI+Targeted therapy	NSCLC	Atezolizumab+Ramucirumab	ORR	II	NCT03689855
	NSCLC	Nivolumab+Anlotinib	ORR	Ib/IIa	NCT04507906
	NSCLC	Camrelizumab+famitinib	OS	III	NCT05106335
	NSCLC	Camrelizumab+Apatinib	PFS	II	NCT04670913
	HCC	Camrelizumab+Apatinib	ORR	II	NCT04826406
	HCC	Sintilimab+Lenvatinib	ORR	II	NCT05010681
	HCC	Pembrolizumab+Regorafenib	ORR	II	NCT04696055
ICI+Targeted therapy	GC/CRC	Tislelizumab+Anlotinib	ORR	II	NCT04777162
	TCC	Pembrolizumab+Ramucirumab	ORR	II	NCT04179110
	RCC	Atezolizumab+Cabozantinib	PFS/OS	III	NCT04338269
ICI+Chemotherapy	NSCLC	Atezolizumab+platinum doublet chemotherapy	ORR	II	NCT03977467
	NSCLC	Pembrolizumab+Docetaxel/Pemetrexed/Gemcitabine	PFS	II	NCT03083808
	SCLC	Durvalumab+Topotecan hydrochloride	OS	II	NCT04607954
	UC	Atezolizumab+Carboplatin+Gemcitabine	PFS	II	NCT03737123
ICI+Radiotherapy	SCCHN	Pembrolizumab+Radiation	ORR	II	NCT03085719
ICI+Other	NSCLC	Atezolizumab+Tocilizumab	ORR	Ib/II	NCT04691817
	Melanoma	Pembrolizumab+4SC-202	safety	Ib/II	NCT03278665
	Solid tumor	Pembrolizumab+BI 1206	safety	I/IIa	NCT04219254

**Table 5** NSCLC, non-small-cell lung cancer; SCLC, small-cell lung cancer; HCC, hepatocellular carcinoma; GC, gastric cancer; CRC, colorectal cancer; UC, urothelial carcinoma; TCC, transitional cell carcinoma; NPC, nasopharyngeal carcinoma; RCC, renal cell carcinoma; SCCHN, squamous cell carcinoma of the head and neck; ICI, immune checkpoint inhibitor; ORR, objective response rate; DCR, disease control rate; PFS, progression free survival; OS, overall survival.

**Table 6:** The clinical data of safety from ICI rechallenge

Cancer type	Rechallenge regimen	≥G3 irAEs No.(%)		IrAEs during ICI rechallenge No.(%)				ORR	DCR	Author
		Prior ICI	Rechallenge ICI	All irAEs	Recurrence of irAEs	Death related to irAE	Cause of death			
NSCLC	Nivolumab	7 (33.3)	1 (4.7)	15 (71.4)	-	-	-	14.3	85.7	Mouri et al^117^
NSCLC	Anti-PD-1±Anti-CTLA-4	13 (34.2)	8 (21)	20 (52.6)	10 (26.3)	2	Pneumonitis/ hepatic failure	47.3	81.5	Santini et al^20^
NSCLC	Anti-PD-(L)1	3 (20)	2 (22.2)	9 (100)	4 (23.5)	1	pneumonitis	5.9	58.8	Kitagawa et al^22^
NSCLC	Anti-PD-(L)1	5 (20.8)	3 (12.5)	4 (16.6)	1 (4.2)	-	-	8.3	45.8	Takahara et al^118^
Melanoma	Anti-PD-1	55 (68.7)	14 (17.5)	40 (50)	14 (17.5)	1	TEN	70	88.7	Pollack et al^109^
Melanoma	Anti-PD-1	58 (86.5)	14 (20.9)	67 (100)	2 (3)	-	-	40	-	Menzies et al^119^
Melanoma	Anti-PD-(L)1	10 (25.6)	6 (15.4)	19 (48.7)	-	-	-	15.4	25.6	Amode et al^122^
Melanoma	Ipilimumab	3 (8)	14 (35)	-	-	1	pneumonitis	10	18	Bowyer et al^120^
Melanoma	Ipilimumab	45 (38.7)	31 (26.7)	54 (46.5)	-	1	colitis	7.7	41.4	Cybulska-Stopa et al^126^
RCC	ICI	18 (26)	11 (15.9)	-	-	-	-	23.4	64	Ravi et al^87^
RCC	Nivolumab+Ipilimumab	3 (6.7)	6 (13.3)	29 (64.4)	-	-	-	20	35.6	Gul et al^83^
Genitourinary cancer	ICI	17 (16)	14 (30)	46 (100)	16 (26.2)	-	-	11	-	Siddiqui et al^107^
Various	Anti-PD-1±Anti-CTLA-4	62 (37.1)	6 (3.6)	57 (34.1)	-	-	-	-	-	Abu-Sbeih et al^121^
Various	ICI	7 (25.9)	5 (18.5)	9 (33.3)	7 (25.9)	-	-	-	-	Bhatlapenumarthi et al^102^
Various	ICI	22 (52.5)	15 (68)	22 (55)	17 (42.5)	-	-	-	-	Simonaggio et al^103^
Various	ICI	7 (17.5)	7 (17.5)	31 (77.5)	19 (47.5)	-	-	-	-	Kartolo et al^110^

**Table 6** NSCLC, non-small-cell lung cancer; RCC, renal cell carcinoma; ICI, immune checkpoint inhibitor; irAE, immune-related adverse events; ORR, objective response rate; DCR, disease control rate; TEN, toxic epidermal necrolysis.

## Abbreviations

ICI: Immune checkpoint inhibitor
irAE: Immune-related adverse events
NSCLC: Non-small-cell lung cancer
PD-1: Programmed cell death 1
PD-L1: Programmed cell death ligand 1
CTLA-4: Cytotoxic T lymphocyte antigen 4
TMB: Tumor mutation burden
NLR: Neutrophil to lymphocyte ratio
LMR: Lymphocyte to monocyte ratio
PLR: Platelet to lymphocyte ratio
PR: Partial response
SD: Stable disease
PD: Progressive disease
OS: Overall survival
PFS: Progression free survival
DCR: Disease control rate
ORR: Objective response rate
OR: Odds ratio
HR: Hazard ratio
ECOG/PS: Eastern Cooperative Oncology Group performance status
BMI: Body mass index
DAMPs: damage associated molecular patterns
PRRs: pattern recognition receptors
